# Supplementary material for: Leveraging supervised learning for functionally informed fine-mapping of cis-eQTLs identifies an additional 20,913 putative causal eQTLs
Source: Nat Commun. 2021 Jun 7;12:3394. doi: 10.1038/s41467-021-23134-8 (PMC8184741; doi:10.1038/s41467-021-23134-8)
Supplement: Supplementary file 3 — Description of Additional Supplementary Files [file 41467_2021_23134_MOESM3_ESM.pdf]

Description of Additional Supplementary Files

File Name: Supplementary Data 1
Description: List of selected feature names and their importances in each tissue

File Name: Supplementary Data 2
Description: List of credible sets where the number of variants became significantly smaller after updating the PIP using EMS as a functional prior in whole blood

File Name: Supplementary Data 3
Description: List of newly identified potentially causal/non-causal eQTLs

File Name: Supplementary Data 4
Description: List of candidate genes identified in co-localization analysis

File Name: Supplementary Data 5
Description: Abbreviation of the assay and the tissue names used in the study

File Name: Supplementary Data 6
Description: Details of grid search for hyperparameter tuning in the training of whole blood EMS

File Name: Supplementary Data 7
Description: List of tissue-specific putative causal eQTLs

File Name: Supplementary Data 8
Description: List of pairs of tissue-specific putative causal eQTLs on same genes

File Name: Supplementary Data 9
Description: Tissue-specific transcription factor (TF) related Basenji feature scores in the corresponding tissue-specific putative causal eQTLs identified by PIP_{EMS}
