# Supplementary material for: Leveraging supervised learning for functionally informed fine-mapping of cis-eQTLs identifies an additional 20,913 putative causal eQTLs
Source: Nat Commun. 2021 Jun 7;12:3394. doi: 10.1038/s41467-021-23134-8 (PMC8184741; doi:10.1038/s41467-021-23134-8)
Supplement: Supplementary file 13 — Reporting Summary [file 41467_2021_23134_MOESM13_ESM.pdf]

nature research

Corresponding author(s): Qingbo Wang / Hilary Finucane

Last updated by author(s): 2021/04/03

Reporting Summary

Nature Research wishes to improve the reproducibility of the work that we publish. This form provides structure for consistency and transparency in reporting. For further information on Nature Research policies, see our Editorial Policies and the Editorial Policy Checklist.

Statistics

For all statistical analyses, confirm that the following items are present in the figure legend, table legend, main text, or Methods section.

n/a	Confirmed	
☐	☒	The exact sample size (*n*) for each experimental group/condition, given as a discrete number and unit of measurement
☐	☒	A statement on whether measurements were taken from distinct samples or whether the same sample was measured repeatedly
☐	☒	The statistical test(s) used AND whether they are one- or two-sided<br>*Only common tests should be described solely by name; describe more complex techniques in the Methods section.*
☐	☒	A description of all covariates tested
☐	☒	A description of any assumptions or corrections, such as tests of normality and adjustment for multiple comparisons
☐	☒	A full description of the statistical parameters including central tendency (e.g. means) or other basic estimates (e.g. regression coefficient) AND variation (e.g. standard deviation) or associated estimates of uncertainty (e.g. confidence intervals)
☐	☒	For null hypothesis testing, the test statistic (e.g. *F*, *t*, *r*) with confidence intervals, effect sizes, degrees of freedom and *P* value noted<br>*Give P values as exact values whenever suitable.*
☐	☒	For Bayesian analysis, information on the choice of priors and Markov chain Monte Carlo settings
☒	☐	For hierarchical and complex designs, identification of the appropriate level for tests and full reporting of outcomes
☐	☒	Estimates of effect sizes (e.g. Cohen's *d*, Pearson's *r*), indicating how they were calculated

Our web collection on statistics for biologists contains articles on many of the points above.

Software and code

Policy information about availability of computer code

Data collection	Code availability<br>Code used in this study is available at https://github.com/FinucaneLab/Expression_ Modifier_Score/

Data analysis	Softwares and packages used for this study:<br>SuSiE v0.8.1.0521 (https://github.com/stephenslab/susie-paper)<br>FINEMAP v1.3.1 (http://www.christianbenner.com)<br>ggseqlogo (https://cran.r-project.org/web/packages/ggseqlogo/index.html)<br>basenji v0.0.1 (https://github.com/calico/basenji)<br>brokenaxis v0.3.1 (https://pypi.org/project/brokenaxes/)<br>joblib v0.11 (https://joblib.readthedocs.io)<br>hail v0.2.26 (https://hail.is)<br>matplotlib v3.2.0 (https://matplotlib.org)<br>numpy v1.18.1 (https://numpy.org)<br>pandas v1.0.1 (https://pandas.pydata.org)<br>scikit-learn v0.21.3 and v0.23.2 (https://scikit-learn.github.io/stable)<br>scipy v1.2.1 (http://scikit-learn.github.io/stable)<br>seaborn v0.9.0 (https://seaborn.pydata.org)<br><br>Code availability<br>Code used in this study is available at https://github.com/FinucaneLab/Expression_ Modifier_Score/

For manuscripts utilizing custom algorithms or software that are central to the research but not yet described in published literature, software must be made available to editors and reviewers. We strongly encourage code deposition in a community repository (e.g. GitHub). See the Nature Research guidelines for submitting code & software for further information.

Data

Policy information about availability of data

All manuscripts must include a data availability statement. This statement should provide the following information, where applicable:

- Accession codes, unique identifiers, or web links for publicly available datasets
- A list of figures that have associated raw data
- A description of any restrictions on data availability

Data availability
EMS for 49 tissues are available at https://www.finucanelab.org/data.

CADD v1.4 and GERP scores were annotated using the hail annotation database (https://hail.is).
ncER scores were downloaded from https://github.com/TelentiLab/ncER_datasets.
DeepSEA v1.0 scores were downloaded from https://humanbase.readthedocs.io/en/latest/deepsea.html
Fathmm v2.3 non-coding scores were downloaded from http://fathmm.bio compute.org.uk
Saturation mutagenesis data was downloaded from the MPRA data access portal (http://mpra.gs.washington.edu).
The raQTL data was downloaded from https://osf.io/w5bzq/wiki/home/.
Human transcription factor (TF) data was downloaded from http://humantfs.ccbr.utoronto.ca/download.php .
The UKBB fine-mapping results are deposited at https://www.finucanelab.org/data .

Field-specific reporting

Please select the one below that is the best fit for your research. If you are not sure, read the appropriate sections before making your selection.

☒ Life sciences ☐ Behavioural & social sciences ☐ Ecological, evolutionary & environmental sciences

For a reference copy of the document with all sections, see nature.com/documents/nr-reporting-summary-flat.pdf

Life sciences study design

All studies must disclose on these points even when the disclosure is negative.

Sample size	We utilized publicly available GWAS/eQTL study results and thus did not pre-determine the human sample sizes.<br>For the enrichment analysis, negative samples (=the number of variant-gene pairs) were randomly downsampled to achieve a total number of variants to be exactly 100,000, to reduce the computational burden while keeping enough number of variants to observe statistical significance. For all the other analysis, we did not pre-determine the sample size (i.e. the sample sizes were defined by the nature of the available data).
Data exclusions	Nothing excluded.
Replication	The subset of the main results where the EMS showed highest enrichment of putative causal eQTLs was replicated in a different cohorts (Biobank Japan and Geuvadis), as noted in supplementary method.
Randomization	Not applicable; we are not performing clinical trial or any related tests.

Blinding	Not applicable; we are not performing clinical trial or any related tests.

Reporting for specific materials, systems and methods

We require information from authors about some types of materials, experimental systems and methods used in many studies. Here, indicate whether each material, system or method listed is relevant to your study. If you are not sure if a list item applies to your research, read the appropriate section before selecting a response.

Materials & experimental systems

n/a	Involved in the study
☒	☐ Antibodies
☒	☐ Eukaryotic cell lines
☒	☐ Palaeontology and archaeology
☒	☐ Animals and other organisms
☐	☒ Human research participants
☒	☐ Clinical data
☒	☐ Dual use research of concern

Methods

n/a	Involved in the study
☒	☐ ChIP-seq
☒	☐ Flow cytometry
☒	☐ MRI-based neuroimaging

Human research participants

Policy information about [studies involving human research participants]

Population characteristics	Population characteristics are described in following articles:<br><br>GTEx: Aguet, F. et al. The GTEx Consortium atlas of genetic regulatory effects across human tissues. bioRxiv 787903 (2019) doi:10.1101/787903.<br><br>UK Biobank: Bycroft, C. et al. The UK Biobank resource with deep phenotyping and genomic data. Nature 562, 203 (2018).<br><br>BioBank Japan: Kanai, M. et al. Genetic analysis of quantitative traits in the Japanese population links cell types to complex human diseases. Nature Genetics 50, 390–400 (2018).<br><br>Geuvadis: Lappalainen, T. et al. Transcriptome and genome sequencing uncovers functional variation in humans. Nature 501, 506–511 (2013).
Recruitment	Recruitment methods are described in the articles above
Ethics oversight	Each study was approved by ethical review, as stated in the articles above

Note that full information on the approval of the study protocol must also be provided in the manuscript.
